# Unique genotype-phenotype correlations within *LAMA2*-related limb girdle muscular dystrophy in Chinese patients

**DOI:** 10.3389/fneur.2023.1158094

**Published:** 2023-05-03

**Authors:** Xiuli Huang, Dandan Tan, Zaiqiang Zhang, Lin Ge, Jieyu Liu, Juan Ding, Haipo Yang, Cuijie Wei, Xingzhi Chang, Yun Yuan, Chuanzhu Yan, Hui Xiong

**Affiliations:** ^1^Department of Pediatrics, Peking University First Hospital, Beijing, China; ^2^Department of Neurology, China National Clinical Research Center for Neurological Diseases, Beijing Tiantan Hospital, Capital Medical University, Beijing, China; ^3^Department of Neurology, Peking University First Hospital, Beijing, China; ^4^Department of Neurology, Research Institute of Neuromuscular and Neurodegenerative Disease, Qilu Hospital, Cheeloo College of Medicine, Shandong University, Jinan, China; ^5^Beijing Key Laboratory of Molecular Diagnosis and Study on Pediatric Genetic Diseases, Beijing, China

**Keywords:** *LAMA2*-related limb girdle muscular dystrophy, LGMD R23, phenotype, genotype, genotype-phenotype correlations

## Abstract

**Background:**

*LAMA2-*related limb girdle muscular dystrophy (LGMD R23) is rare. The detailed clinical phenotypes and genetic information associated with LGMD R23 are unknown.

**Methods:**

We conducted a retrospective cross-sectional and longitudinal study on 19 LGMD R23 patients.

**Results:**

Normal early motor development was observed in 84.2% patients. Mild orthopedic complications were observed in 42.1% patients. 36.8% patients had seizures, which is unusually frequent in LGMD. Epilepsy was eventually diagnosed in 26.3% patients. 46.7% patients presented with motor neuropathy. Genetic analysis identified 29 pathogenic variants, with missense and frameshift variants being the most common. The mutant sites were mainly distributed in the N-terminal and G-like domains of laminin. The missense variants are distributed near the N-terminus (exons 3–11), whereas frameshift variants are distributed in exons 12–65. Five patients were diagnosed with epilepsy and all of them harbor at least one missense variants in exon 4. 71.4% variants of patients with motor neuropathy located in the LN domain.

**Conclusions:**

Missense variants in exon 4 maybe correlated with epilepsy and variants in the LN domain maybe correlated with motor neuropathy in Chinese patients. Our study expands the clinical and genetic spectrum caused by *LAMA2* variations and provides novel genotype-phenotype correlations of LGMD R23.

## 1. Introduction

*LAMA2-*related muscular dystrophy (*LAMA2*-MD) is an autosomal recessive muscular dystrophy caused by mutations in LAMA2 gene (OMIM ^*^156,225). *LAMA2* located on chromosome 6q22–23, encodes the α2 chain (also called merosin) of the heterotrimeric extracellular matrix (ECM) protein laminin-211, a major component of the basal membrane (BM) (1, 2). Laminin-211 is broadly expressed and mainly in skeletal muscle, but it is also expressed in the brain (astrocytes at the blood-brain barrier, pericytes, ventricular-subventricular zones), Schwann cells, synaptic basal lamina of peripheral nerves, the heart, and skin tissues. The α2 chain is involved in extracellular matrix architecture, cellular attachment, neurite growth, and migration of Schwann cells (3–5). The receptors for laminins include integrins, dystroglycan and heparan sulfate proteoglycans (3, 6). Integrins are transmembrane glycoprotein receptors and dystroglycan is a transmembrane receptor of the Dystrophin-Glycoprotein Complex (DGC). The receptor α-Dystroglycan (αDG) has been found to play a more substantial role in mediating BM anchorage to the myofiber as compared to integrin (3, 6).

Two clinical phenotypes of *LAMA2*-MD have been identified: early-onset *LAMA2* related congenital muscular dystrophy (*LAMA2*-CMD, also known as congenital muscular dystrophy type 1A, MDC1A) and mild, late-onset limb girdle muscular dystrophy (LGMD R23) (7–9). The majority of *LAMA2*-CMD patients have complete laminin-α2 deficiency on muscle biopsy, whereas in the majority of mild cases a partial laminin-α2 deficiency has been documented (2). The *LAMA2*-MD patients have some same characters, such as white matter changes (WMC) in brain detectable by magnetic resonance imaging and some patients have peripheral nerve involvement (2). *LAMA2*-CMD is a common form of CMD and accounts for 24–37% of all congenital muscular dystrophies (1, 8, 10). It is characterized by congenital hypotonia, delayed motor development, progressive muscle weakness affecting the shoulder and pelvic girdles leading to the loss of independent ambulation and joint contractures. Brain magnetic resonance imaging (MRI) showed widespread WMC (11, 12).

LGMD R23 is rare. In Denmark, the LGMD corresponding to a *LAMA2*-mutation frequency was 2.3% in LGMD cohorts (10). In an Italian cohort, the relative frequency of LGMD R23 among all LGMD patients was 1.3% (13). Of 506 patients with inherited neuromuscular disorders (NMD) in the Lebanese population, only eight patients were LGMD R23 (14). Neither detailed clinical features nor genotypes of LGMD R23 were described in these cohorts. There are a few small case reports to date (7, 15), but the detailed clinical phenotypes and genetic information associated with LGMD R23 are still largely unknown. The prevalence of Chinese LGMD R23 is currently unknown. Here, we describe a relatively large cohort of Chinese patients carrying homozygous or compound heterozygous mutations of *LAMA2* who manifest a limb girdle muscular dystrophy phenotype. Our purpose was to describe the detailed clinical features and the genetic variations of LGMD R23, and to establish the possible genotype-phenotype correlations within this group of patients.

## 2. Materials and methods

### 2.1. Patients

This is a retrospective cross-sectional and longitudinal study conducted on LGMD R23 patients. All patients were under clinical follow-up at the pediatric department of Peking University First Hospital in the period between June 2013 and December 2022. The diagnosis of LGMD R23 was established based on clinical manifestation, age of symptom onset, and molecular genetic analyses. All participants met the following inclusion criteria: (1) age of onset after 1 year (10, 16) and achieved independent walking before 2 years of age (9); (2) one of the following clinical manifestations: exhibiting proximal muscle weakness, elevated creatine kinase (CK) levels, seizures, or abnormal white matter signal on brain MRI; (3) molecular genetic analyses identified homozygous or compound heterozygous mutations in *LAMA2*. The exclusion criteria were as follows: (1) age of onset before 1 year, (2) atypical clinical presentation of LGMDs (for example, distal weakness at symptom onset), and (3) genetic and/or histopathological findings suggestive of other neuromuscular diseases. This study protocol was approved by the Ethics Committee of Peking University First Hospital [No. 2015(916), Beijing, China]. Informed consent was obtained from all participants or their guardians.

### 2.2. Clinical and pathological studies

Clinical data were collected which included age of onset of symptoms, motor function, orthopedic complications, and CNS involvement. Accessory examinations including electroencephalogram (EEG), brain MRI, and electromyography (EMG), were analyzed if performed. Muscle biopsies were performed on patients who could not definitive diagnosis by genetic testing, and frozen sections (6 μm) were processed for routine histological and immunohistochemical staining. The mouse monoclonal antibodies against laminin-a2 we used was MAB 1922 (100 L; 5H2, Merck Millipore, Darmstadt, Germany, Lot #3187395).

### 2.3. *LAMA2* variants analysis

Genomic DNA was isolated from peripheral blood samples of patients and their parents, as well as from their affected siblings. The subtle pathogenetic variation and copy number variation (CNV) within all coding and adjacent intronic sequences of *LAMA2* were analyzed using whole exome sequencing (WES), multiplex ligation-dependent probe amplification (MLPA), or bi-directional Sanger sequencing confirmation in different laboratories. Sequence variants were described based on the Human Genome Variation Society guidelines for mutation nomenclature (17). The pathogenicity of novel missense variants was evaluated using the variant-classification guidelines of the American College of Medical Genetics and Genomics (ACMG) and determined based on population frequency, *in silico* prediction programs, and reports in locus-specific databases.

### 2.4. Statistical analysis

Statistical analyses were performed using SPSS (version 25) software. The Shapiro-Wilk test was used to test the normality of the measurement data. Non-normally distributed data are expressed as medians (ranges). Normally distributed date are expressed as mean ± standard deviation. Categorical data are expressed as percentage (%). Fisher's exact test was used as appropriate.

## 3. Results

Nineteen patients from 17 non-consanguineous families were identified (12 males, 7 females) in this study. The median followed-up time was 4.5 years (range 1.5–9.5 years). Clinical data are summarized in Supplementary material 1, and genetic data are summarized in Table 1.

**Table 1:** Genetical analysis of patients with LGMD R23.

**Patient**	**Exon**	**Domain**	**Nucleotide change**	**Predicted amino acid change**	**Parental derivation**	**Variation type**
P1	4	LN	c.437C>A	p.Ser146Tyr	M	MS
	IVS4	LN	c.640-1G>C		P	Splicing
P2	62	G-like	c.8844_8845insAAGGCTC	p.Phe2949Lysfs^*^21	*De novo*	FS
	12	IV	c.1732_1736del	p.Leu578Alafs^*^30	M	FS
P3	4	LN	c.437C>A	p.Ser146Tyr	P	MS
	27	IV	c.4048C>T	p.Arg1350^*^	M	NS
P4	5	LN	c.830C>T	p.Ser277Leu	P	MS
	4	LN	Exon4del		M	CNV
P5	56	G-like	c.7927del	p.Arg2643Glufs^*^7	*De novo*	FS
	61	G-like	c.8815C>T	p.Gln2939^*^	M	NS
P6	9	EGF-like	c.1300C>T	p.Arg434^*^	P	NS
	11	EGF-like	c.1544G>A	p.Cys515Tyr	M	MS
P7	47	G-like	c.6634_6645del	p.Ser2212_G2215del	P	Del
	3	LN	c.332A>C	p.Gln111Pro	M	MS
P8, P9	36-65	α I	Exon36-65del		P	CNV
	10	EGF-like	c.1358G>C	p.Cys453Ser	M	MS
P10	IVS7	EGF-like	c.1027+3A>G		M	Splicing
	IVS17	EGF-like	c.2450+4A>G		P	Splicing
P11	3	LN	c.332A>C	p.Gln111Pro	M	MS
	IVS58	G-like	c.8244+5G>C		P	Splicing
P12	38	αI	c.5476C>T	p.Arg1826^*^	M	NS
	13	IV	c.1793_1795del	p.Val598del	P	Del
P13	65	G-like	c.9311dup	p.Asn3104Asnfs^*^39	P	FS
	14	IV	c.2049_2050del	p.Arg683Serfs^*^21	M	FS
P14	22	EGF-like	c.3149del	p.Gly1050Alafs^*^25	P	FS
	59-63	G-like	Exon59-63del		M	CNV
P15	4	LN	c.443G>A	p.Arg148Gln	*De novo*	MS
	43	α II	c.6235del	p.Thr2079Argfs^*^24	M	FS
P16, P17	4	LN	c.437C>T (hom)	p.Ser146Phe	P, M	MS
P18	4	LN	c.439C>T (hom)	p.Pro147Ser	P, M	MS
P19	4	LN	c.437C>A	p.Ser146Tyr	M	MS
	51	G-like	c.7186delG	p.Gly2396fs^*^3	P	FS

ACMG, American College of Medical Genetics and Genomics; α I, laminin helical coiled-coil domain I; α II, laminin helical coiled-coil domain II; CNV, copy number variation; Del, short in frame deletion; FS, frameshift mutation; hom, homozygous; IV, laminin IV type A1 or A2; IVS, intervening sequence (intron); LN, laminin N-terminal; M, maternal; MS, missense mutation; NS, nonsense mutation; P, paternal; Splicing, splicing mutation; VUS, variant of uncertain significance.

### 3.1. Onset of symptoms

**Figure 1:**
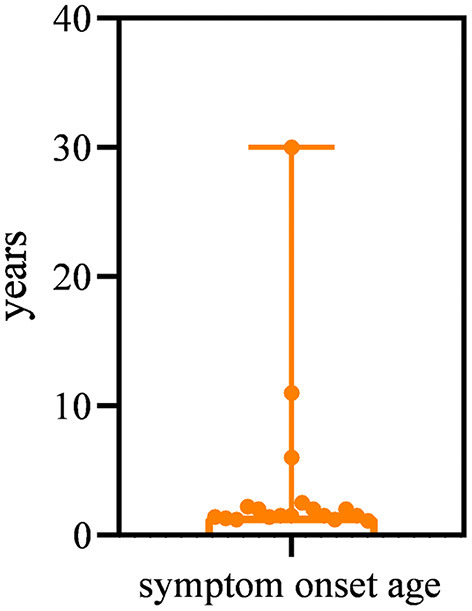
Symptom onset age of LGMD R23 patients.

All patients had a disease onset spanning between 13 months and 30 years, with a median age of 1.5 years. The majority (15/19, 78.9%) of patients had symptom onset by the age of 3 years (Figure 1). The most common presentation (13/19, 68.4%) was abnormal gait and frequent falls in the first 3 years of life. Three patients (15.8%) presented with difficulty in running and jumping started at age 2 years, 2.5 years, and 6 years respectively. One patient (P19) manifested weakness of the lower limbs of unknown cause at 10 years of age. However, the other two patients (P16, P18) first visited the neurology clinic because of epilepsy at 11 years and 30 years of age, respectively. Of these two patients, one (P16) patient had normal gait but her running speed slower than normal children of the same age. The other patient (P18) showed fatigue at the age of 37 years.

### 3.2. Gross motor function and orthopedic complications

All patients achieved independent ambulation at a median age of 18 months (range 13–20 months). Sixteen patients (84.2%) achieved normal early motor milestones. Three patients (P5, P10, and P14) had mild delay achieving independent ambulation at 19 months, 19 months, and 20 months of age, respectively. All but one of the 19 patients were ambulatory at the last evaluation (P14 gradually lost ambulation at 5.5 years of age).

Eight patients (8/19, 42.1%) suffered orthopedic complications, such as joint contracture, scoliosis, and talipes varus, and 4 patients (21%) exhibited more than one of the above symptoms. Joint contractures were observed in four patients (21%) at a median age of 6 years (range 1.5–10 years). One patient (P14) showed contractures of both ankles and knees, whereas the other patients only exhibited contractures of the ankles. Two patients required Achilles tendon lengthening surgery at the age of 9 years and 10 years, respectively. Spine deformity was observed in 4 patients (21%). Two patients presented with mild lordosis at the ages of 3.5 years and 22 years and two patients exhibited mild scoliosis at the ages of 7 years and 10 years, respectively. Talipe varus was reported in four patients (21%), and tightened Achilles tendons were observed in three patients.

### 3.3. Biochemical test results

Creatine kinase (CK) levels were moderately increased. The average value for maximum CK was 1,766.0 ± 1,110.8 U/L (range between 342 and 4362 U/L).

### 3.4. Central nervous system involvement

#### 3.4.1. Seizures

Seizures are common presentations of LGMD R23, and 36.8% (7/19) of the patients had seizures during the period of data collection. Two patients had family histories of epilepsy (P14's grandfather's brother and P18's uncle). No epilepsy-related genes were found in whole exome sequencing of these seven patients with seizures. The median age at seizure onset was 14 years (range 1.8–31 years). Epilepsy was diagnosed in five patients (26.3%). Two patients (10.5%, 2/19) developed seizures after fever at the ages of 1.8 years and 2.6 years, and were diagnosed with febrile seizures. Patients diagnosed with epilepsy developed seizures at a median age of 24 years (range 11–31 years). The seizure type in the two patients with FS was generalized tonic–clonic seizures (GTCS). Focal-onset seizures with impaired awareness were detected in all five patients (100%) who were diagnosed with epilepsy. Two of them also had seizures with preserved awareness. Generalized onset seizures were observed in one patient with tonic–clonic seizures (P19) and one with atypical absence seizures (P17). Visual aura was described in one patient (P16, blurred vision and flashing). Autonomic signs and vomiting were reported in one patient (P18).

EEG reports were available for all seven patients diagnosed with seizures. The most observed occurrences in our cohort were interictal EEG paroxysms with focal and posterior localization, and bilateral or unilateral temporal or temporo-occipital epileptiform abnormalities. One patient (P16) also exhibited sharp waves in the left central area. One patient (P18) had bilateral frontal epileptiform abnormalities. One patient (P14) with FS had a normal EEG. Although P13 showed left temporal epileptiform abnormalities and had five fever-induced seizures with six episodes between 2.6 years and 4 years of age, at the time of data collection, she was 7 years old and experienced no seizures for 3 years without any antiepileptic drugs and was diagnosed with complex febrile seizure. EEG studies were also performed in four of the other 12 patients without seizures, and no abnormalities were found.

Five patients with epilepsy received antiepileptic treatment. Two patients (40%, 2/5) had long-term seizure control (more than 18 months seizure-free at the conclusion of the study) with oxcarbazepine monotherapy. Two patients (40%, 2/5) showed a partial benefit with combination therapy (P17 with topiramate transition to levetiracetam plus sodium valproate, P18 with phenytoin plus oxcarbazepine). One patient experienced recurrent seizures from the onset of epilepsy despite being treated with a combination of levetiracetam and oxcarbazepine then transition to levetiracetam and sodium valproate.

#### 3.4.2. Cognition

Eighteen of the 19 patients (94.7%) exhibited normal cognition. P18 had normal cognition before the age of 30 years. He started school at 5 years of age, with average academic performance. He graduated from vocational secondary school and was able to hold a regular job. Since the onset of seizures at the age of 30 years, he has experienced memory deterioration. He had a Mini-Mental State Examination (MMSE) score of 27 and a Montreal Cognitive Assessment (MoCA) score of 21 at the age of 35 years, indicating mild cognitive impairment (MCI).

#### 3.4.3. Neuroimaging findings

Eighteen patients had brain MRI scans, with 83.3% (15/18) showing widespread abnormal white matter hyperintensities on T2-MRI, and 16.7% (3/18) showing mild white matter changes predominately in the anterior and/or posterior horns of the lateral ventricle. Occipital pachygyria was found in two patients (11.1%, 2/18). One of them had refractory epilepsy, and the other had sudden death but without seizure.

### 3.5. Peripheral nervous system involvement

EMG was used to assess peripheral nervous system involvement. Fifteen patients underwent EMG studies, and almost half of the patients (46.7%, 7/15) showed neuropathy, mainly presenting with motor neuropathy. Motor nerve conduction velocities were decreased in all seven patients with peripheral neuropathy, three (20%, 3/15) of them also had a reduction in compound motor action potential (CMAP) amplitudes. Two patients (13.3%, 2/15) presented with prolonged median nerve latency. The H-reflex was not detected in one patient (P19). Sensory nerve involvement with decreased conduction velocity was observed in two patients (13.3%, 2/15).

### 3.6. Cardiac and respiratory involvement

The pulmonary function test of one patient (P7) showed moderate-to-severe restrictive lung disease at 8.7 years of age as previously described (17); however, no patients reported symptoms of breathing difficulty in our current cohort. Electrocardiography (ECG) and/or echocardiography (ECHO) data were available for 17 patients (ECG for 12, ECHO for 14). ECG results showed sinus arrhythmia in three patients at age of 3.4 years, 3.6 years, and 26 years, respectively, and one patient exhibited left anterior fascicular block. Abnormal left ventricular diastolic function was detected in one patient; the remaining ECHO results showed normal function or mild valvular regurgitation.

### 3.7. Survival

The current cohort has a median age of 10 years (range 3.2–37 years). All patients were living at the conclusion of this study except one who died at 14 (P8) years old from unknown cause.

### 3.8. Genetic characteristics

**Figure 2:**
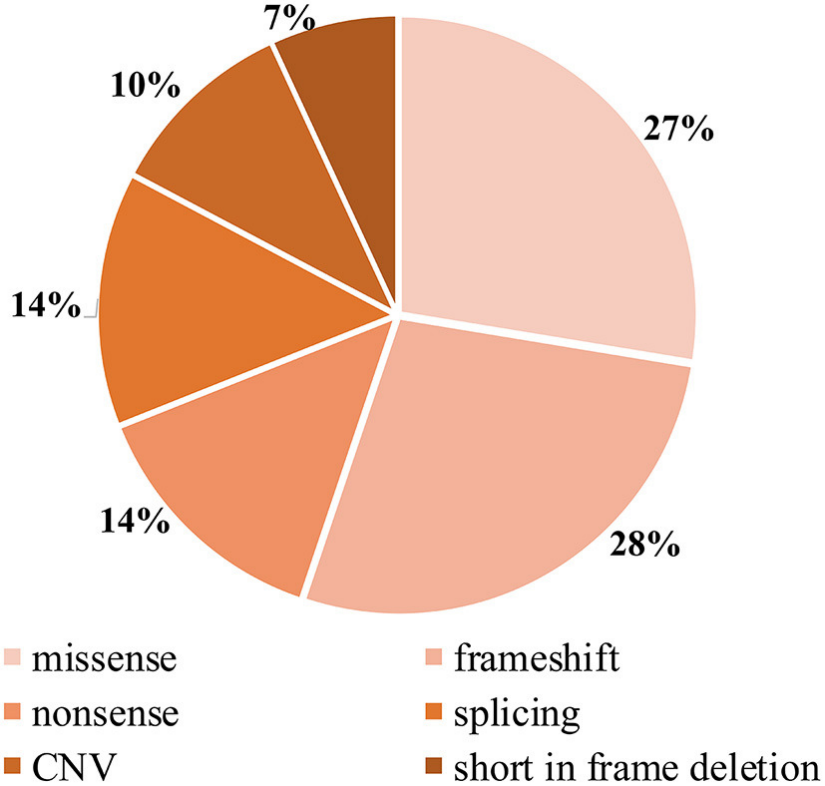
Distribution of types of *LAMA2* mutations found in our patients.

Detailed genetic analysis are summarized in Table 1 and **Figure 4**. A total of 29 pathogenic variants were identified (three novel and 26 known) (according to the LAMA2 gene homepage—Global Vario me shared LOVD and https://www.ncbi.nlm.nih.gov/clinvar/?term=LAMA2[gene]) in the 34 different alleles from 19 patients (17 families) that comprised our cohort. These 29 variants include eight missense, eight frameshift (six deletion, one duplication, one insertion), four nonsense, four splice site, three large copy number variations (CNV), and two short in frame deletion of several nucleotides (Figure 2). The variant c.437C>A (p.Ser146Tyr) was a common pathogenic variant in Han Chinese patients (*n* = 3). Missense variant was identified in 13 chromosomes, and 61.5% (8/13) were located in exon 4.

Among the known pathogenic variants, 23 have been described in our previous publications (17–22), and two splice site variants (c.2450+4A>G, c.1027+3A>G) and a small nucleotide deletion (c.1793_1795del, p.Val598del) have been reported in the literature (2). Of the three novel variants, one was splice site variant (c.8244+5G>C), one was a single nucleotide deletion (c.7186delG, p.Gly2396fs^*^3) leading to frameshift and a premature termination codon, and the last was a homozygous missense variant (c.439C>T, p.Pro147Ser) from two non-consanguineous parents. According to the ACMG variant-classification, c.8244+5G>C is classified as variant of uncertain significance (VUS), and c.7186delG is classified as pathogenic variant.

**Figure 3:**
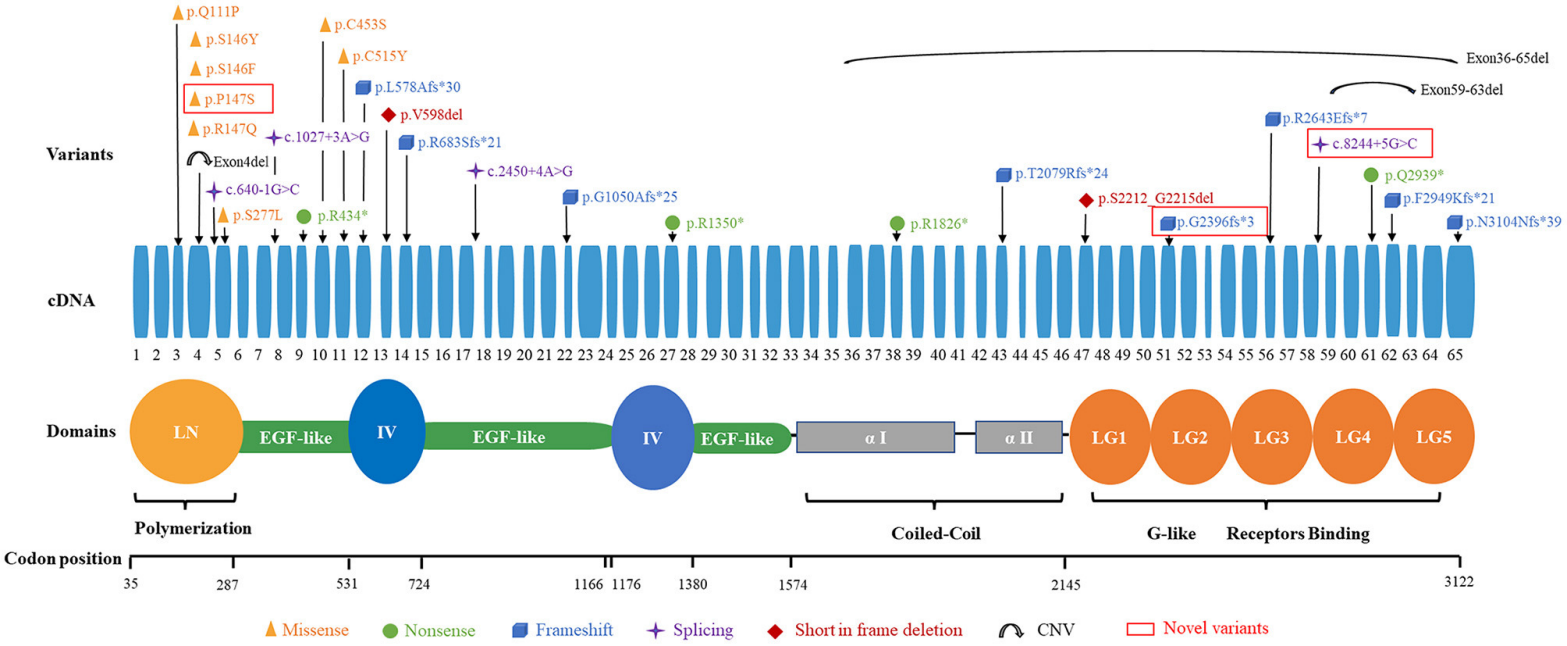
Schematic of the *LAMA2* gene and variants found in our patients.

The 29 disease-causing variants (except CNVs) were mainly distributed in the N-terminal (LN; 7/29, 24.1%), G-like domain (LG; 7/29, 24.1%), EGF-like domain (6/29, 20.7%), and domain IV (4/29, 13.8%) of laminin. The missense variants were mainly located in the LN domain and adjacent supporting EGF-like domains of laminin-α2. These variants were distributed in exons 3–11, with half of them in exon 4. Comparatively, eight frameshift variants were located in exons 12–65 (Figure 3).

### 3.9. Pathogenicity analysis of novel variants

**Figure 4:**
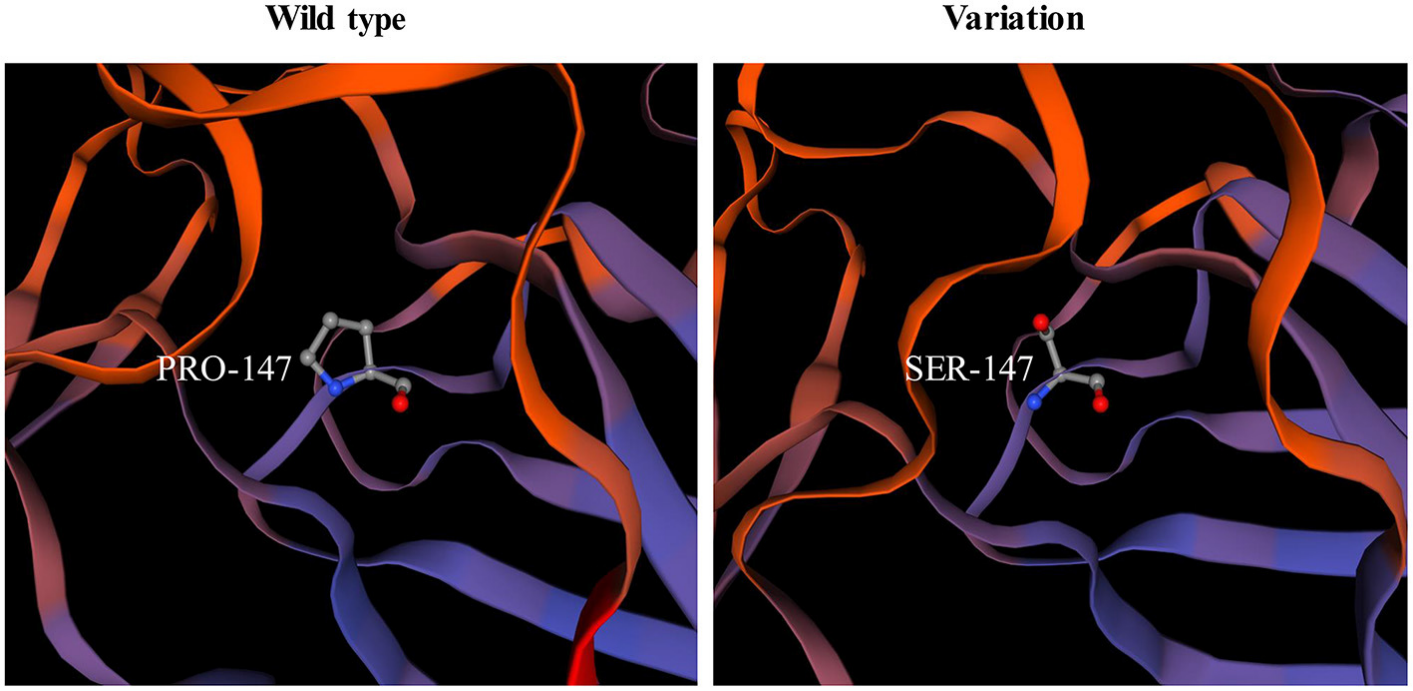
p.P147S is predicted by modeling three-dimension structure of the protein.

**Figure 5:**
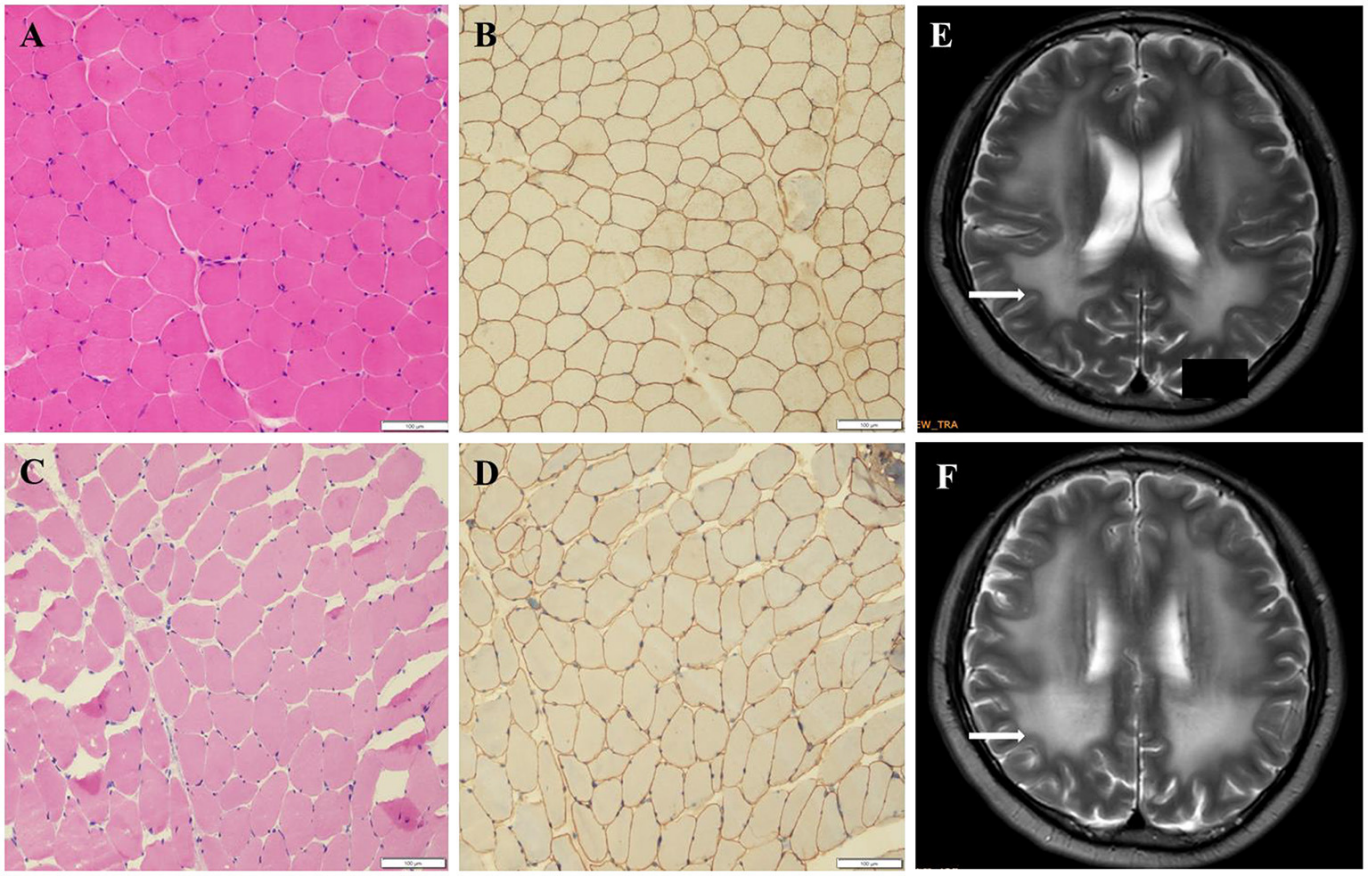
Muscle biopsy and brain MRI of P18 with homozygous missense variant (p.P147S). **(A)** Hematoxylin-eosin staining (HE) from the control. **(B)** Immuno-labeling for merosin from the control. **(C)** The HE staining from P18 showed increased variations in fiber size and nuclear trans location. **(D)** Immuno-labeling for merosin showed nearly normal expression in all myofibers compared to the control. **(E, F)** The brain MRI showed widespread abnormal white matter hyperintensities on T2.

The pathogenicity of novel missense variants (c.439C>T, p.Pro147Ser) was evaluated. This variant c.439C>T was not found in the gnomAD and the 1000 Genomes Project database, and was predicted to be damaging using four software programs [Combined Annotation Dependent Depletion (CADD), PolyPhen-2 HumVar, SIFT PROVEAN Protein, and Mutation Taster, the score was 25, 1.0, −7.49 and 0.9999, respectively]. This amino acid substitution was predicted to be damaging by modeling the three-dimensional (3D) structure of the protein (Figure 4). Due to this patient CK level was mild increased, but still had no limb weakness at 35 years old, muscle biopsy was performed to further verified the pathogenicity of this variant. Muscle biopsy showed mild increased variations in fiber size and internalized nucleus. The expression of laminin alpha 2 in all myofibers was nearly normal (Figures 5A–D). The patient had clinical manifestations of LGMD R23, such as increased CK levels, epilepsy, typical widespread abnormal white matter hyperintensities on T2-MRI found in *LAMA2*-MD (Figures 5E, F), and peripheral neuropathy. Therefore, c.439C>T was identified as a pathogenic variant and this patient (P18) was diagnosed as LGMD R23. At the age of 37 years, P18 manifested fatigue and the diagnosis of LGMD R23 was further confirmed.

### 3.10. Genotype-phenotype correlations

Five patients (P15–P19) diagnosed with epilepsy were identified from four families. All of them harbor at least one mutant variant in exon 4. Three of the patients had homozygous missense variants in exon 4 (6 alleles), and the other two patients had compound heterozygous variants with one missense variants in exon 4 and the other in another exon (exon 43 and 51, respectively). Therefore, among the 10 chromosomes from these 5 patients with epilepsy, 8 of them harbored missense variant in exon 4 (8/10, 80%). In contrast, of the 14 patients without epilepsy, only 2 missense variants in exon 4 were found in their 28 chromosomes (2/28, 7.1%). This difference is statistically very significant (*P* < 0.0001). We also analyzed 14 chromosomes from 7 patients with motor neuropathy that harbored mutant variants and found 10 of them (10/14, 71.4%) harbored a mutant variant in the LN domain. In contrast, eight patients (16 alleles) without peripheral neuropathy (confirmed by EMG), only 2 (2/16, 12.5%) variants were located in the LN. This difference is also statistically very significant (*P* = 0.0022).

## 4. Discussion

To the best of our knowledge, this is the first cohort of patients with LGMD R23. LGMD is a group of genetic disorders that primarily affect proximal skeletal muscles, leading to progressive weakness in the limb girdles and dystrophic changes in muscle histology. In 2018, the 229th ENMC International workshop revised the LGMD nomenclature and classification. To date, more than 30 different LGMD subtypes listed in the current classification system are mainly autosomal recessive (AR) (9, 23). The most common LGMDs are calpainopathy (LGMD R1), dystroglycanopathies (multiple subtypes, including LGMD R9), dysferlinopathy (LGMD R2), sarcoglycanopathies (LGMD R3–R6), and anoctaminopathy (LGMD R12) (24). In China, the most common subtypes are LGMD R1 and LGMD R2 (25, 26). The prevalence of LGMD R23 in China is currently unknown. In 2017, Yu et al. reported a Chinese LGMD cohort. In this cohort, two patients had *LAMA2* mutations, two patients had *COL6A2* mutations, and one patient had *COL6A1* mutations with LGMD characteristics. These five patients were not included in the LGMD cohort because they had not been placed on the LGMD subtype list until 2018. However, we estimated that the relative frequency of LGMD R23 among all patients with LGMD was 1.8% in the Chinese population (26). In our previous study, the proportion of LGMD R23 phenotypes among all *LAMA2-*related muscular dystrophies was 10.8% (18).

In our cohort, patients onset age ranging from 13 months to 30 years old. In 2011, Gavassini et al. reported a LGMD patient due to *LAMA2* mutation whose onset age was 59 years old (16). These indicate the onset age of LGMD R23 was highly variable, similar to other LGMD subtypes (24). Most patients had normal early motor milestones, and orthopedic complications were mild and less frequent than those of *LAMA2*-CMD (18). In our study, most patients were ambulatory at last evaluation. Lokken et al. (10) reported a 69 years old patient and Magri et al. (7) reported a 75 years old patient, both of them were ambulatory. These suggest that the progression of LGMD R23 may be slow. The thigh muscle quantitative MRI in our four patients showed fatty infiltration predominantly affecting the gluteus maximus, adductor magnus, and long head of the biceps femoris as our previously described (27). CNS involvement is a remarkable characteristic of LGMD R23. The comorbid rate of seizures and epilepsy in LGMD R23 is higher than that in other LGMD subtypes and in the general population. Focal-onset seizures with impaired awareness and paroxysmal discharge with focal and posterior localization in interictal EEG are the most common presentations. The comorbid rates of seizures and epilepsy among the early-onset *LAMA2*-CMD patients is 9.5% and 7.8%, respectively (18). For the patients of Duchenne muscular dystrophy (DMD), some clinical manifestations similar with LGMD, the prevalence of epilepsy ranges 3.1–12.3%, and absence seizures, GTCS, and focal seizures are common (28, 29). Some dystroglycanopathies patients also manifested LGMD, in our previous study, 15.1% of patients had seizures due to dystroglycanopathies, most of whom were diagnosed with febrile seizures, and GTCS was common (30). For the general pediatric population, the prevalence of epilepsy was 0.5–1% (29). Other LGMD subtypes that coexist with seizures or epilepsy have rarely been described. An important diagnostic feature of LGMD R23 is the brain MRI. Abnormal white matter hyperintensity on T2-weighted images is typical findings. We also found that 10.5% of patients had occipital pachygyria. Patients with DMD showed normal brain MRI. In early onset dystroglycanopathies, brain structural abnormalities were variable, and cortical dysplasia (polymicrogyria or pachygyria) and infratentorial malformations (cerebellar cysts, vermis and/or hemispheric hypoplasia/dysplasia, and/or brainstem hypoplasia) were observed (31). Brain MRI changes and/or brain malformations were rarely found among the other LGMD subtypes.

The mechanisms of LGMD R23 and the other two types of DGC-related muscular dystrophies (DMD and dystroglycanopathies) with an increased incidence of epilepsy are not well understood. One hypothesis is that laminin and DGC impact inhibitory synapses and that mutations can alter neuronal activity (3). In a Spanish cohort, it was found that the extension of polymicrogyria in *LAMA2*-RD may serve as a predictor of epilepsy occurrence (5). However, no LGMD R23 patients with polymicrogyria were found in our study. Among the two patients with occipital pachygyria (OP), one had refractory epilepsy and the other had sudden death at 14 years of age. OP may be associated with adverse outcomes in Chinese patients.

Peripheral nerve involvement is also a remarkable characteristic of LGMD R23. In our study, nearly half patients presenting with decreased motor nerve conduction, suggesting that peripheral demyelinating neuropathy is a disease feature. Motor neuropathy could be a potentially detrimental contributor to disease burden. It is possible that the reason of limb weakness in some LGMD R23 patients were largely due to peripheral neuropathy rather than muscle involvement.

Cardiovascular and respiratory system impairments were uncommon in our patients, however, they were observed in LGMD R4, R5, and R6 (24). Due to the development of next-generation sequencing technology and injury, muscle biopsy in our cohort was not a routine examination. In our study, immuno-labeling for laminin-α2 in three patients showed nearly normal expression, consistent with literature reported that LGMD R23, the mild phenotype of *LAMA2*-MD is generally associated with partial reduction in laminin-α2 (2).

In previously reported cases, almost all patients with LGMD R23 had one in-frame mutation (mainly was missense variant) (7). In our cohort, the proportion of patients with one in-frame mutation in the 17 families was 70.6% (12/17) and missense variant was found in 64.7% (11/17) families. Missense variants and frameshift variants were the most prevalent in our cohort. Mutant alleles were mainly distributed in the LN and LG of laminin. Interestingly, in our cohort, the missense variants are located near the N-terminus (exons 3–11), and frameshift variants are distributed in exons 12–65. The high-frequency pathogenic variant in LGMD R23 is c.437C>A (p.Ser146Tyr), which differ from Han Chinese *LAMA2*-CMD patients (18).

In the current study, missense variants located in exon 4 were more prevalent in patients with epilepsy. This genotype-phenotype correlations between missense variants in exon 4 and epilepsy may exist. Two patients (P1 and P3) who harbored missense variants in exon 4 were still young. Whether they would manifest seizure need long term follow up. However, this relationship between missense variants in exon 4 and epilepsy were not found in non-Chinese patients. In 2020, Magri et al. reported five Italy LGMD R23 patients and performed a wide PubMed search of the literature concerning LGMD R23 (7). In this study, twenty-six patients were reported and a total of 20 non-Chinese LGMD R23 patients had genetic results. Among the 8 epilepsy patients, no patients harbored missense variant in exon 4. Of the 12 patients without epilepsy, five missense variants in exon 4 were found in their 24 chromosomes (5/24, 20.8%), but there was no statistical difference (*P* = 0.0712). We analyzed 16 chromosomes from 8 patients with epilepsy, and found 4 of them (4/16, 25%) harbored missense variants in exon 5. In contrast, there were no patients harbored missense variant in exon 5 without epilepsy. This difference is statistically significant (*P* = 0.0199) (7). This difference suggests that there are ethnic differences in the phenotype and genotype features between Chinese and non-Chinese patients. There seems to be a genotype-phenotype correlation between the genetic variants distributing in LN region and the tendency of being affected with motor neuropathy in Chinese patients. Due to LGMD R23 was very rare and the cohort was small, this correlation was indeterminate.

This study had several limitations. First, although this is the largest case series of LGMD R23, the number of samples was not substantial. Clinical and genetic characteristics based on limited data and natural history studies with a longer time would be more adequate. Second, important assessments were not performed in this retrospective study. For example, quantitative muscle MRI was only performed in four patients due to the cost and long testing time, we could not analysis the MRI data in this study, the progressive patterns of muscle degeneration were not clear. Motor function scale assessments were not performed in our patients. We describe patients with orthopedic complications; the exact progressive degree and speed of muscle weakness and orthopedic involvement are not well known.

In conclusion, our study provides valuable information on the clinical and genetic features and genotype–phenotype correlations of LGMD R23. The onset age of LGMD R23 is highly variable and the progression of LGMD R23 may be slow. In addition to the muscle phenotype, attention should be paid to nervous system involvement. White matter abnormality, high incidence of seizures and motor neuropathy were common features of LGMD R23. Brain MRI should be performed to all DGC-related muscular dystrophies, even without CNS symptom, because it can address the diagnosis. Having more time available for further study, collecting more clinical and genetic data, and performing motor function scale assessments are necessary to understand the natural history of LGMD R23 and management of this disease.

## Data availability statement

The data presented in the study are deposited in the GenBank repository, accession number OQ865237.

## Ethics statement

This study protocol was approved by the Ethics Committee of Peking University First Hospital [No. 2015(916), Beijing, China].

## Author contributions

XH: follow-up, data analysis, and manuscript writing. DT, LG, and JD: data collection. JL: histological and immunohistochemical staining. ZZ, YY, and CY: data provision. CW and XC: muscle biopsies analysis. HX: study design and manuscript revision. All authors participated in drafting and critically revising the article and approved the final manuscript.
