# Supplementary material for: Unique genotype-phenotype correlations within LAMA2-related limb girdle muscular dystrophy in Chinese patients
Source: Front Neurol. 2023 May 3;14:1158094. doi: 10.3389/fneur.2023.1158094 (PMC10190595; doi:10.3389/fneur.2023.1158094)
Supplement: Supplementary file 1 [file Table_1.DOCX]

**Supplemental Material 1** Clinical features of patients with LGMD R23

| Patient/ sex/age | Age at onset | Symptoms of onset | motor milestone  (age) | Contracture  (age) | Spinal deformity (age) | Talipes varus  (age) | CK U/L (age) | Seizure/seizure type(seizure onset age) | EEG changes | EMG changes | Brain MRI (age) | EEG/ECHO changes | Others |
| --- | --- | --- | --- | --- | --- | --- | --- | --- | --- | --- | --- | --- | --- |
| P1/M/5.2y | 1.2 y | abnormal gait | HC(3m), S (7m), W (14m) | No | No | No | 1344 (2 y) | No | Na | MNCV and MNCMAPA reduced | WMH  (2 y) | Normal/Normal | Thigh muscle MRI: diffuse fatty infiltration |
| P2/M/8y | 1.5 y | abnormal gait | HC (3 m), S (6m), W (18m) | No | No | No | 4362 (3.2 y) | No | Na | Myopathic changes | WMH (3.4 y) | Normal/Normal |  |
| P3/M/8.3y | 1.1 y | abnormal gait | HC (2 m), S (6m), W (13m) | No | No | No | 709  (8 m) | No | Normal | Myopathic changes | WMH (3.6 y) | Sinus arrhythmia /Na |  |
| P4/F/8.4y | 1.5 y | abnormal gait | HC (3 m), S (6m), W (18m) | No | No | Yes(2.3y) | 1337 (2 y) | No | Normal | Myopathic changes, MNCV reduced | WMH (1.7 y) | Normal/ Na | Thigh muscle MRI: diffuse fatty infiltration |
| P5/M/8y | 1.5 y | abnormal gait | HC (3 m), S (8m), W (19m) | No | No | No | 2000 (5 y) | No | Na | Normal | Anterior and posterior horn of lateral ventricle (3.6 y) | Na /Na | Thigh muscle MRI: diffuse fatty infiltration, Mild reduction of merosin (2.3 y) |
| P6/F/10.5y | 1.5 y | abnormal gait | HC (4 m), S (6m), W (18m) | No | No | No | 3103 (1 y) | No | Na | Myopathic changes | Na | Na /Normal |  |
| P7/F/13y | 2 y | abnormal gait | HC (3 m), S (6m), W (18m) | Ankle(1.5y) | Scoliosis (10 y) | Yes(1.5y) | 1018 (6 y) | No | Na | Myopathic changes, MNCV and MNCMAPA reduced | WMH (5.7 y) | Normal/Normal |  |
| P8/M/14 y | 6 y | difficulty running and jumping | HC (4 m), S (10m), W (16m) | No | No | No | 2103 (6.4 y) | No | Na | Myopathic changes | Posterior horn of lateral ventricle, OP (7.8 y) | Normal/Mild TR | normal expression of merosin(8y); Died at 14y |
| P9/F/11.5y | 1.2 y | abnormal gait | HC (4 m), S (7m), W (14m) | No | No | No | 1481 (1 y) | No | Na | Myopathic changes | Posterior horn of lateral ventricle (2.5 y, 8.9 y) | Normal /Normal | Case 8’s younger sister |
| P10/M/3.2y | 1.4y | abnormal gait | HC (3 m), S (6m), W (19m) | No | No | No | 1935  (1.5y) | No | Normal | Myopathic changes | WMH  (1.4 y) | Normal/Mild TR |  |
| P11/M/4.8y | 1.4y | abnormal gait | HC (4 m), S (6m), W (17m) | No | Lordosis(3.5y) | Yes(3.5y) | 846  (3.4y) | No | Na | Myopathic changes | WMH  (3.3 y) | Na/Na |  |
| P12/M/4.7y | 2.2y | abnormal gait | HC (3 m), S (6m), W (13m) | No | No | No | 1248  (3.3y) | No | Normal | Na | WMH  (3.3 y) | Sinus arrhythmia /PFO | Thigh muscle MRI: mild edema |
| P13/F/7.5y | 2.5 y | difficulty running and jumping | HC (3 m), S (6 m), W (13m) | No | No | No | 2419  (2.8y) | febrile seizure/GTCS (1.8y ) | Epileptic discharge in left temporal region | Na | WMH (1.8 y) | Normal/PFO |  |
| P14/F/10y | 2 y | abnormal gait | HC (3 m), S (6 m), W (20m) | Knee, ankle(6y) | Scoliosis (7y) | Yes(6y) | 3078 (2.5y) | febrile seizure/GTCS (2.6 y) | Normal | Myopathic changes, MNCV and MNCMAPA reduced | WMH (1.6 y, 2.3 y) | Normal/ Normal | Loss of W (5.5 y)  Grandfather's brother had epilepsy |
| P15/M/25y | 1.3 y | abnormal gait | HC (3 m), S (6m), W (16m) | No | No | No | 3609  (15y) | Epilepsy, F-IA (14 y) | epileptic discharge in right occipital and posterior temporal region | Na | WMH, (18 y) | Na/ Mild TR |  |
| P16/F/20y | 11y | epilepsy | HC (4 m), S (6 m), W (18m) | No | No | No | 442 (13y) | Epilepsy,  F-A/F-IA (11 y) | epileptic discharge in left central ，bilateral occipital and posterior temporal region | Na | WMH, OP (13.2 y) | Normal/Na |  |
| P17/M/29y | 2 y | difficulty running and jumping | HC (4 m), S (9 m), W (18m) | Ankle(6y) | Lordosis(22y) | No | 1025 (21 y) | Epilepsy, F-IA, atypical absences (24y) | multifocal discharge, mainly in bilateral temporal region | MNCV reduced | WMH (22 y) | LAFB,  Sinus arrhythmia /Mild MR, TR | Case 16’s elder brother |
| P18/M/37y | 30y | epilepsy | Normal* | No | No | No | 342  (35y) | Epilepsy, F-A/F-IA (30y) | epileptic discharge in bilateral frontal, occipital and temporal region | MNCV, SNCV and MNCMAPA reduced, latency prolonged | WMH  (35y) | Na/ left ventricular diastolic function decreased | MCI;  Patient’s uncle had epilepsy; normal expression of merosin (35 y); |
| P19/M/35y | 10y | weakness of lower limbs | Normal* | Ankle(10y) | No | No | 1154  (34y) | Epilepsy, F-IA,GTCS(31y) | epileptic discharge in left temporal region | MNCV, SNCV and MNCMAPA reduced, Latency prolonged,  No H-reflex | WMH  (34y) | Na/Normal |  |

CK: creatine kinase; ECG: electrocardiogram; ECHO: echocardiography; EMG: electromyogram; F: female; F-A: Focal seizure without impaired awareness; F-IA, focal seizure with impaired awareness; GTCS: general tonic-clonic seizure; HC: head control; LAFB: left anterior fascicular block; M: male; m: months; MCI: mild cognitive impairment; MNCV: motor nerve conduction velocity; MNCMAPA: motor nerve compound muscle action potential amplitude; MR: mitral regurgitation; Na: not available; OP: occipital pachygyria; PFO: patent foramen ovale; RRI: recurrent respiratory tract infection; S: sitting; SNCV: sensory nerve conduction velocity; TR: tricuspid regurgitation; W: walking; WMH: abnormal white matter hyperintensities; y: years; *:the exactly time was unknown.
